# Increased Tropism of Extracellular Vesicles Derived from Palmitic Acid-Treated Hepatocytes to Activated Hepatic Stellate Cells

**DOI:** 10.3390/membranes12101023

**Published:** 2022-10-21

**Authors:** Momoka Yamaguchi, Takanori Kanazawa, Sumire Morino, Shingo Iioka, Yuta Watanabe, Naoki Dohi, Kenjirou Higashi, Hiromu Kondo, Tomohisa Ishikawa

**Affiliations:** 1 Department of Pharmacology, School of Pharmaceutical Sciences, University of Shizuoka, 52-1 Yada, Suruga-ku, Shizuoka 422-8526, Japan; 2 Department of Pharmaceutical Engineering and Drug Delivery Sciences, School of Pharmaceutical Sciences, University of Shizuoka, 52-1 Yada, Suruga-ku, Shizuoka 422-8526, Japan; 3 Laboratory of Pharmaceutical Technology, Graduate School of Pharmaceutical Sciences, Chiba University, 1-8-1, Inohana, Chuo-ku, Chiba 260-0856, Japan

**Keywords:** extracellular vesicles, liver fibrosis, hepatic stellate cells, hepatocytes, palmitic acid, drug delivery system

## Abstract

Myofibroblast-like activated hepatic stellate cells (aHSCs), which produce collagen, a major cause of liver fibrosis, are specific target cells for antifibrotic treatment. Recently, several reports have indicated that extracellular vesicles (EVs) play important roles in cell-to-cell communication through their tropism for specific cells or organs. Therefore, the present study aimed to identify aHSC-directed EVs by focusing on cell-to-cell interactions in the liver under pathological conditions. EVs were derived from the hepatocyte cell line AML12 treated with or without palmitic acid (PA) and evaluated for their physical properties and uptake by the aHSC cell line LX-2. AML12-derived EVs had a mean particle diameter of 110–130 nm, negative charge, and expressed the exosomal makers CD9 and CD63. PA-treated AML12 cells released larger EVs with higher protein levels than those without PA treatment. The intracellular uptake efficacy of EVs derived from PA-treated AML12 cells into activated LX-2 cells was significantly higher than those without PA treatment. Our study revealed that PA treatment induces hepatocytes to release EVs with aHSC-tropism. These findings may contribute to the development of an EV-based drug delivery system (DDS) for aHSC-targeted therapy and provide new insights into the role of steatotic hepatocyte-derived EVs in physiological or pathophysiological functions.

## 1. Introduction

Liver fibrosis is a major consequence of chronic liver disease, in which excessive deposition of extracellular matrix is driven by activation of hepatic stellate cells (HSCs) [1]. HSCs are non-parenchymal cells that exist in the space of Disse between hepatocytes and sinusoidal endothelial cells, and account for approximately 10% of the total number of liver-resident cells [2,3,4]. In liver fibrosis, activated HSCs (aHSCs) are the main source of extracellular matrix, especially collagen, which is produced by transforming growth factor-β (TGF-β) stimulation [2]. Moreover, the reversion of aHSCs to inactivated ones has been reported to occur during spontaneous healing [3]. Thus, aHSCs are likely to play a critical role in the progression of liver fibrosis.

Several reports have shown the involvement of signaling pathways in the regulation of HSC activation, especially the reversion of aHSCs to quiescent state-HSCs (qHSCs) via in vivo analyses [4,5]. However, in vivo analyses are susceptible to off-target effects on hepatocytes that account for approximately 70% of the total number of liver-resident cells. Hence, a drug-delivery system (DDS), which avoids transfer to hepatocytes and specifically targets aHSCs, is essential for therapeutic intervention in liver fibrosis.

Extracellular vesicles (EVs), such as exosomes (30–200 nm) and microvesicles (200–1000 nm), play important roles in cell-to-cell communication [6,7,8]. Recently, several miRNA-encapsulating EVs derived from hepatocytes or liver stem cells were reported to contribute to the regulation of HSC activation [9]. Integrins of tumor-secreted EVs have been shown to mediate organ-specific targeting, and initiate the establishment of pre-metastatic niche formation, which is likely to redirect the metastasis of tumor cells that lack the capacity to metastasize to a specific organ [10]. This study suggests that EVs play an important role in the tropism of specific cells and organs. Moreover, EVs have been reported to be associated with physiological functions, the development of various diseases, and control of the cellular microenvironment [8,9,11]. However, the target cell- or organ-tropism of EVs remains to be fully elucidated.

The fibrotic microenvironment in the liver is controlled by the various liver-resident cells [12,13]. In particular, hepatocytes, which constitute a large population of liver cells, have been reported to interact with HSCs [11,14,15]. Furthermore, the lipotoxic treatment of hepatocytes affects HSC activation [9,14]. Based on these reports, we hypothesized that the release of EVs with aHSC-tropism, which is closely related to the progression of liver disease, is enhanced under pathological conditions. Therefore, the present study aimed to identify aHSC-targeted EVs by focusing on cell-to-cell interactions in the liver under pathological conditions. EVs were derived from the hepatocyte cell line AML12 treated with or without palmitic acid (PA) and evaluated for their physical properties and uptake efficiency into the activated HSC cell line LX-2. In addition, comprehensive gene expression analysis was performed using mRNA sequencing to investigate the genes that were affected by PA treatment of AML12 cells.

## 2. Materials and Methods

### 2.1. Cell Culture

Human HSC line LX-2 cells (P25–35) [16] were purchased from Merck (Darmstadt, Germany, #SCC064) and cultured in high-glucose Dulbecco’s modified Eagle’s medium (DMEM; Sigma, St. Louis, MO, USA) containing 2% fetal bovine serum (FBS; Biowest, Nuaillé, France) as previously described [17]. Non-transformed murine hepatocyte line AML12 cells (P39-42) [18] were obtained from the American Type Culture Collection (ATCC, Rockville, MD, USA, #CRL-2d254™) and maintained in DMEM/Ham’s F-12 supplemented with 10% FBS, 10 μg/mL insulin, 5.5 μg/mL transferrin, 6.7 ng/mL selenium (ITS-G Supplement, FUJIFILM Wako Pure Chemical Corporation, Osaka, Japan, #090-06741), and 40 ng/mL dexamethasone (MP Biomedicals, Irvine, CA, USA, #194561). The culture medium was replaced with serum-free medium containing the above supplements to induce hepatocyte differentiation. Both LX-2 and AML12 cells were seeded in culture dishes or multi-well plates and incubated overnight in a 95% air-5% CO_2_ at 37 °C.

### 2.2. Preparation of PA

PA was dissolved at 250 mmol/L in 50% ethanol/high-purity water and diluted with 10% bovine serum albumin (BSA) to prepare a 10 mmol/L PA stock solution. The PA stock solution was then loaded with N_2_, aliquoted, and stored at −20 °C until use.

### 2.3. Oil-Red-O Stain

AML12 cells cultured in the presence or absence of PA (200 μM) for 48 h were fixed with 2% paraformaldehyde in phosphate-buffered saline (PBS) for 15 min and treated with 0.18% Oil-red-O stain (FUJIFILM Wako Pure Chemical Corporation, Osaka, Japan)/60% isopropanol in ultra-pure water for 20 min at room temperature (around 25 °C).

### 2.4. mRNA-Sequencing Analysis of AML12 Cells

Total RNA was extracted from AML12 cells using the NucleoSpin RNA Kit (MACHEREY-NAGEL GmbH & Co. KG, Duren, Germany) as previously described [19]. Library construction and sequencing were performed by Novogene (Beijing, China). RNA quality was assessed using a 5400-fragment analyzer system (Agilent Technologies, Tokyo, Japan). The libraries were sequenced on a DNBSeq-T7 platform (MGI Tech Co. Ltd., Shenzhen, China) and paired-end reads were generated. The read sequences were aligned to the *Mus musculus* (GRCm38.p6) reference genome using hisat2 (ver. 2.0.5). Differential expression genes (DEG) of the two conditions/groups (two biological replicates per condition) were detected using the DESeq2 R package (1.20.0). Gene ontology (GO) enrichment analysis of DEG was performed using the clusterProfile R package. GO terms with corrected values of *p* < 0.05 were considered significantly enriched by DEG. We used the gene set enrichment analysis (GSEA) tool (http://www.broadinstitute.org/gsea/index.jsp accessed on 20 September 2022). The GO and Reactome datasets were independently used for GSEA. All raw and mRNA-sequencing data in the present study have been listed in the DDBJ Sequence Read Archive under accession number DRA #014859.

### 2.5. Isolation of EVs from AML12 Cells

AML12 cells were differentiated by culturing in DMEM/Ham’s F-12 without FBS for 2–4 days and then treated with or without PA for 48 h. The cells were then further cultured in serum-free medium in 100 mm dishes for four days. The conditioned medium (approximately 130 mL) was filtered through 0.22 μm membrane filters after removing the cells by centrifugation (2000× *g*, 10 min). The supernatant was concentrated to 10 mL using Amicon Ultra-15 Centrifugal 30 K filter units (Merck, Darmstadt, Germany). EVs were collected using size-exclusion chromatography (SEC). After removing the storage solution, 700 μL of exosome-depleted FBS (Gibco A2720801, Lot No. 2243287p) was added to a size-exclusion column (EVSecond L70, GL Sciences Inc., Tokyo, Japan), and then 4.5 mL of PBS filtered with 0.22 μm membrane filters was run through the column. Concentrated culture medium samples were diluted to 1.5 mL with filtered PBS, and the sample was overlaid on the size-exclusion column followed by elution with 100 μL of filtered PBS per fraction. The flow-through was collected in 100 µL fractions, and fractions 1–9 were combined for characterization and intracellular uptake analysis.

### 2.6. Characterization of EVs

Isolated EVs were stored at 4 °C and characterized within seven days of isolation. Cryo-transmission electron microscope (TEM) observations of EVs were performed using a JEM-2100F (JEOL Co., Ltd., Japan) at 120 kV below −170 °C using a Gatan 626 cryo-holder (Gatan Inc., Pleasanton, CA, USA) in liquid nitrogen. For the preparation of EVs samples, 2 μL of the EVs suspension was dropped onto a collodion membrane-supported grid Cu200 (Nissin EM Corporation, Tokyo, Japan) with hydrophilic treatment 60 s using HDT-400 (JEOL Ltd., Tokyo, Japan) and rapidly frozen in liquid ethane at approximately −170 °C following the removal of excess solution. The particle diameter distribution and concentration of EVs were obtained using a nanoparticle-tracking analysis system (NanoSight; Quantum Design, San Diego, CA, USA). The protein concentration of EVs was determined using bicinchoninic acid (BCA) assay. The amount of protein per particle was calculated by dividing the protein concentration of the EVs by their particle concentration. The zeta potential (ζ potentials) of the EVs was measured using a Zetasizer Ultra (Malvern Panalytical, Malvern, UK).

### 2.7. Western Blot Analysis of EVs

Western blot analyses of EVs were performed as our previously described [20]. Following 8% SDS-polyacrylamide gel electrophoresis, the proteins were transferred to a polyvinylidene difluoride membrane and blocked with 2% BSA in TBS-T buffer for 1 h at room temperature. The membrane was incubated with anti-CD9 (1:1000, ABclone, Tokyo, Japan, #A19027) or anti-CD63 (1:1000, ABclone, #A5271) antibodies overnight at 4 °C. After washing off the free antibodies, the membrane was then incubated with HRP-conjugated anti-rabbit IgG antibody (1:5000, Sigma-Aldrich, St. Louis, MO, USA #A6154) for 1 h at room temperature. Signals were detected with ImmunoStar LD Reagents (Fujifilm Wako Pure Chemical Corporation, Osaka, Japan) using Amersham Imager 680 QC (Cytiva, Logan, UT, USA).

### 2.8. Intracellular Uptake of EVs in Hepatocytes and HSCs

AML12 and LX-2 cells were seeded at a density of 3 × 10^4^ cells/well in 6-well plates. AML12 cells were treated with serum-free medium for 4 days for differentiation into hepatocytes. The LX-2 cells were treated with TGF-β1 for 48 h for activation. EVs were labeled with the ExoSparkler Exosome Membrane Labeling Kit-Deep Red (Dojindo, Kumamoto, Japan, #EX03). The cells were washed twice with PBS and then loaded with EVs with an equal amount of protein (3 ng proteins/mL) for 4 h at 37 °C with 95% air-5% CO_2_. After 4 h, the cells were observed using an all-in-one fluorescence microscope BZ-X810 (Keyence, Osaka, Japan). The medium was discarded, and the cells were collected by 0.025% trypsin and fixed with 10% paraformaldehyde in PBS for 15 min. The intracellular uptake of EVs was measured by flow cytometry (FACS Celesta multi-color cell analyzer, Becton Dickinson, Franklin Lakes, NJ, USA) as in our previous study [19,20]. The deep red fluorescence intensity of the cells was analyzed using flow cytometry. The transfected cells were detected by subtracting the autofluorescence. Cells were analyzed by acquiring 10,000 events and the intracellular uptake efficiency was calculated based on the percentage (%) of deep-red positive cells among all collected cells.

### 2.9. Statistics

Data are expressed as the mean ± standard error of mean (S.E.M.). Groups were compared using Student’s *t*-test. Statistical significance was set at *p* < 0.05.

## 3. Results

### 3.1. Isolation of EVs Derived from AML12 Cells

**Figure 1:**
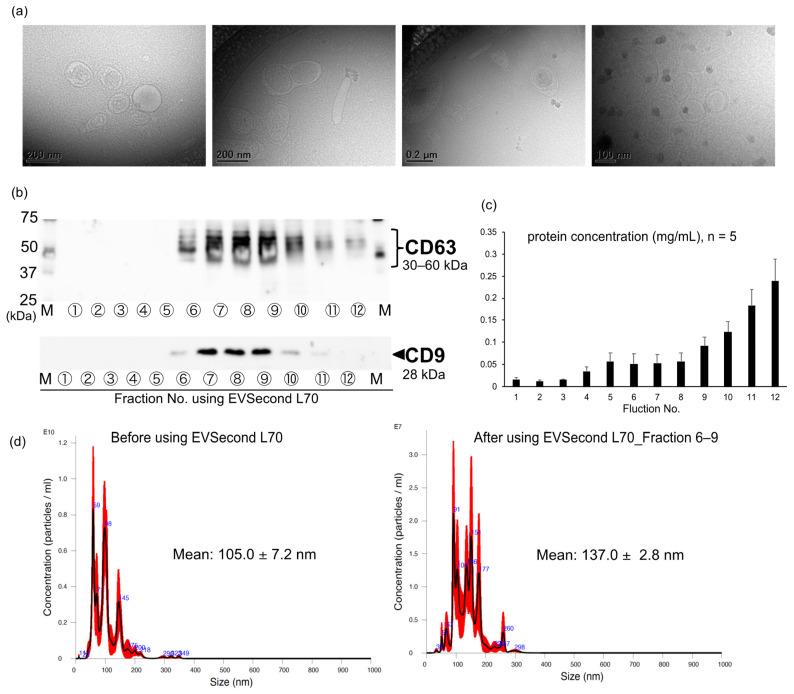
Evaluation of the physical properties of EVs derived from AML12 cells. (**a**) Cryo-TEM images of EVs derived from AML12 cells. Particles indicated by arrows are crystal ice (artifacts) [21]. (**b**) Western blot analysis of CD63 and CD9 in EVs collected from fractions 1–12 using size-exclusion chromatography (SEC; EVSecond L70). (**c**) Protein concentration in fractions 1–12 collected using SEC. Data are expressed as mean ± S.E.M. (*n* = 5). (**d**) Averaged finite track length adjustment (FTLA) concentration and size determined by nanoparticle tracking analysis (NTA) of EVs before and after purification using SEC. Error bars indicate ± standard error of the mean.

Cryo-TEM revealed vesicle-like EVs of various sizes and morphologies with lipid bilayers and vesicular internal structures derived from AML12 cells (EV-AMLs) (Figure 1a). The protein concentration of the fractions increased in a fraction number-dependent manner, and increased markedly after fraction 9 (Figure 1b). The exosomal markers CD9 and CD63 were detected in the EV-AMLs in fractions 6–11 and fractions 6–12, respectively, by SEC (Figure 1c), suggesting that the EV-AMLs collected in this study may be exosome-like EVs. Furthermore, the EV-AMLs had the negative charge characteristic of exosomes with ζ-potential values of −11.8 ± 3.3 mV (*n* = 4), which supports this notion. The mean particle diameter of the EV-AMLs collected in fractions 6–9 using SEC was slightly larger than that in all the other fractions (Figure 1d). Furthermore, analysis of particle size distribution showed that purification with SEC reduces the number of particles with sizes < 100 nm and sharpens the distribution (Figure 1d).

### 3.2. PA Induced the Accumulation of Fatty Acids in AML12 Cells

**Figure 2:**
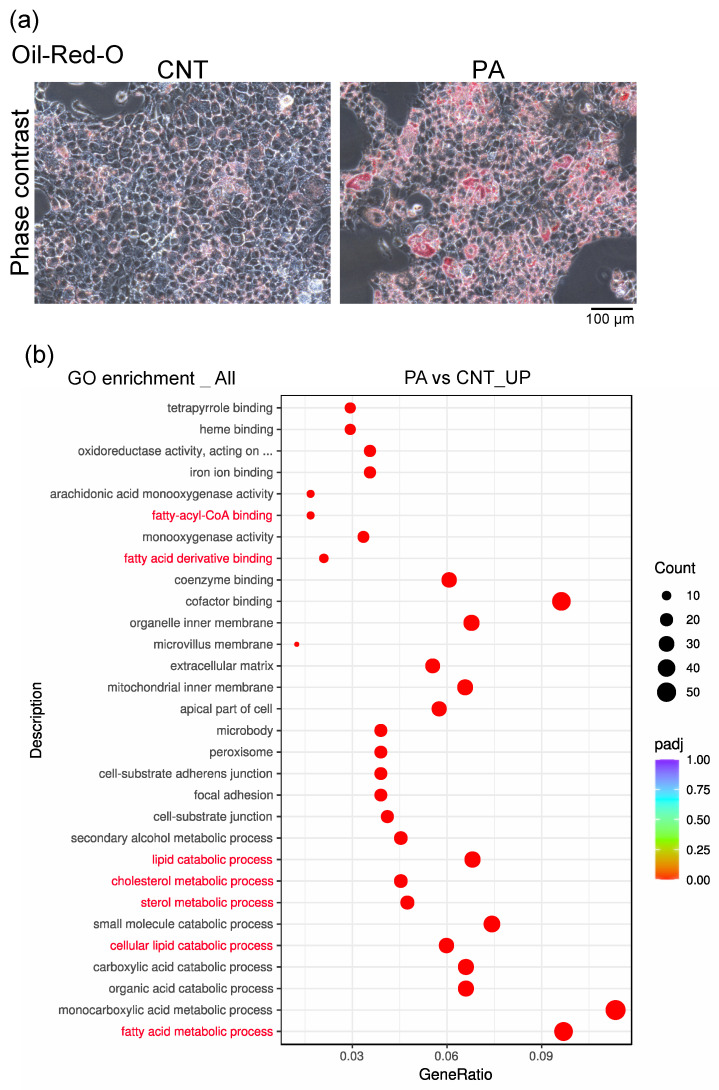
PA induces the accumulation of fatty acids in AML12 cells. (**a**) Oil-red-O staining of AML12 cells treated with or without PA (CNT). Typical phase contrast images of Oil-red-O-stained lipid droplets are shown. (**b**) Gene ontology (GO) enrichment analysis using mRNA-sequencing. Dot plots of the top 30 upregulated differentially expressed genes in PA vs. CNT are shown.

The accumulation of fatty acids, a major source of steatohepatitis [22], was markedly increased in AML12 cells following treatment with PA (200 μM; AML^PA^) for 48 h (Figure 2a). Therefore, we performed a comprehensive gene expression analysis using mRNA sequencing in AML12 cells treated without (AML^CNT^) or with PA. The differentially expressed genes that were upregulated in AML^PA^ compared to AML^CNT^ were enriched in gene ontology (GO) categories related to fatty acids, such as fatty acid metabolic process (GO:0006631), cellular lipid catabolic process (GO:0044242), lipid catabolic process (GO:0016042), and fatty acid derivative binding (GO:1901567) (Figure 2b). These results suggest that PA promotes fatty acid accumulation in AML12 cells.

### 3.3. Characterization of EV-AML

**Figure 3:**
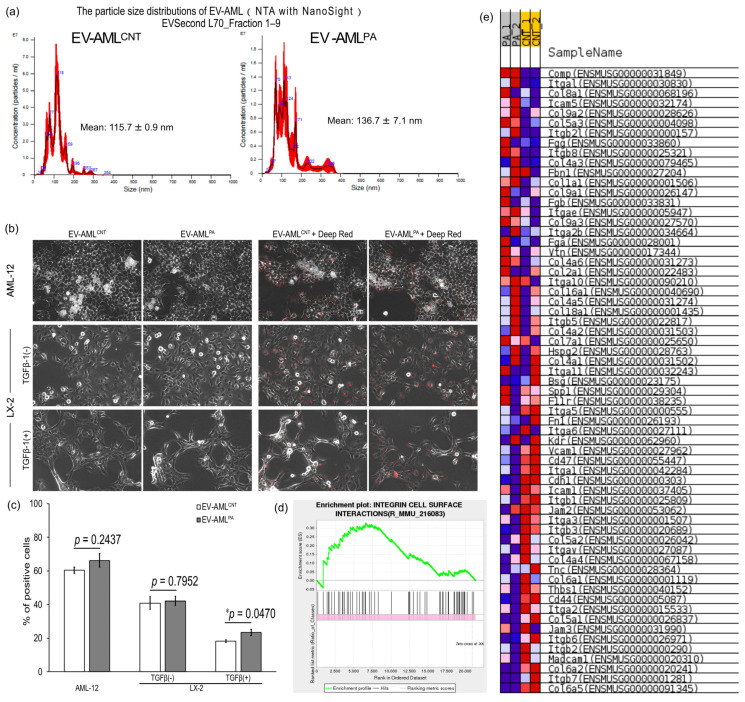
Intracellular uptake of EV-AML into AML12, quiescent or activated LX-2 cells, and the related genes affected by the PA treatment in AML12. (**a**) Particle size distribution of EV-AML^CNT^ and EV-AML^PA^ was detected using nanoparticle tracking analysis (NTA) with NanoSight. Finite track length adjustment concentration was used to determine particle size. (**b**) Phase contrast images of AML12 cells and LX-2 cells treated with or without TGF-β1 merged with images of fluorescent-unlabeled (**left**) or labeled (**right**) EV-AML^CNT^ and EV-AML^PA^. (**c**) Intracellular uptake efficiency of each EV-AML was calculated based on percent of deep red fluorescence-positive cells detected by FACS. Data are expressed as mean ± S.E.M. (*n* = 4). * *p* < 0.05 by Student’s *t*-test. (**d**) Enrichment plot of integrin cell surface interactions (R_MMU_216083)-associated (normalized enrichment score [NES] = +1.3987197) genes. Positions of gene set members in the rank-ordered list: (**e**) Blue–pink o’gram of the analyzed gene set (R_MMU_216083).

EV-AML^CNT^ and EV-AML^PA^ purified by SEC showed mean particle diameter of 115.7 nm and 136.7 nm, respectively, and the distribution of the particle diameters had multiple peaks, indicating that the EV-AMLs comprise a population with heterogeneous particle sizes (Figure 3a). The protein levels in EV-AML^CNT^ and EV-AML^PA^ were 7.191 × 10^−9^ μg/particle and 15.596 × 10^−10^ μg/particle, respectively (Table 1). These results suggest that AMLs treated with PA release EVs with higher protein levels than those without PA treatment.

### 3.4. Intracellular Uptake of EV-AML into AML12, and Quiescent or Activated LX-2 Cells

Finally, the intracellular uptake of EV-AML^CNT^ and EV-AML^PA^ into AML12, quiescent LX-2, and activated LX-2 cells was examined. AML12 and the two LX-2 cells were observed 4 h following the application of unlabeled or fluorescence-labeled EV-AML to the cells (Figure 3b,c). Among these cells, AML12 cells showed the highest uptake of EV-AML. The uptake of EV-AML^PA^ was significantly higher than that of EV-AML^CNT^ into activated LX-2 cells, but not into AML12 cells or quiescent LX-2 cells (Figure 3c). Exposure to either EV and fluorescently labeled EVs did not affect the morphology of AML12 and either LX-2 cells (Figure 3b). Based on these results, the mRNA-seq data of AML12 cells were reanalyzed, and the integrin cell-surface interactions’ gene set (R_MMU_216083) was found to be enriched in PA-treated AML12 cells (normalized enrichment score (NES) = +1.3987197) (Figure 3d,e).

## 4. Discussion

The present study showed that the intracellular uptake efficiency of AML12 cell-derived EVs into activated LX-2 cells, but not into quiescent LX-2 cells or AML12 cells, was increased following PA treatment of AML12 cells. This suggests that the treatment of hepatocytes with PA induces the release of EVs with aHSC- tropism. These results may help develop EV-based DDS as a definitive therapy for liver fibrosis.

Hepatocyte-derived EVs have been shown to be incorporated into HSCs [9,23]. Interestingly, the amount of EVs derived from hepatocytes was shown to be reduced due to phagocytosis by Kupffer cells during the progression of NASH, suggesting that the amount of EV uptake into HSCs varies according to the disease progression [11]. However, there have been no reports on their uptake into aHSCs. In the present study, we demonstrated, for the first time, that EVs secreted from PA-treated hepatocytes show increased aHSC-tropism. The results of oil-red-o staining and GO enrichment analysis (Figure 2) suggested that PA induces steatosis in AML12 cells. PA stimulates EV production by renal tubular epithelial cells [24]. Moreover, lipid-induced toxicity in hepatocytes increases the release of EVs, which induces an inflammatory macrophage phenotype [25] and promotes a fibrotic response in HSCs [9]. Exposure to PA is known to induce steatosis and lipotoxicity in hepatocytes [26]. Taken together, PA-induced steatosis in hepatocytes is not only likely to increase the amount of EVs released from the hepatocytes and alter EV contents, but also to affect the EV tropism.

Integrins in tumor-secreted EVs have been reported to determine organ specificity [10]. A previous report showed that EVs expressing integrin αVβ5 specifically bind to Kupffer cells and mediate liver tropism, whereas that of EVs expressing integrin α6β4 and integrin α6β1 regulate lung tropism by bind to lung-resident fibroblasts and epithelial cells [10]. ECM proteins, Integrins, or glycolipids on EVs allow for them to bind to recipient cells expressing the suitable receptors on their surfaces [27,28]. High expression of integrin αVβ3 [29] or α8β1 [30] has been reported in aHSCs. Moreover, fibronectin and integrin α5 have been shown to colocalize on the cell surface of aHSC and myofibroblasts [31]. The integrin family of cell adhesion receptors recognize the tripeptide motif Arg-Gly Asp (RGD motif) contained in collagens, fibronectin, laminin, vitronectin, thrombospondin, and osteopontin, which are the principal components involved in ECM interactions and important adhesive molecules [32,33]. Genes associated with ‘integrin cell surface interactions’ (R_MMU_216083) were found to be enriched in PA-treated AML12 cells (Figure 3d,e). In addition, PA induced the release of larger EVs with higher protein amounts (Table 1). Taken together, compared to EV-AML^CNT^, EV-AML^PA^ may express high levels of integrins and their ligands that easily bind to aHSCs.

EVs isolated using SEC have been reported to be relatively pure and functional, and SEC is a reproducible, scalable, and inexpensive method that does not require specialized equipment or user expertise [34]. EVs are categorized as exosomes (30–200 nm diameter) or microvesicles (200–1000 nm size) based on their size [6,7,8], have a negative charge that allows for interaction with positively charged molecules [35], and express exosomal markers such as CD9 and CD63 [6,7,8]. The present study indicated that EVSecond L70 enables the removal of proteins and purification of EVs with a mean particle size of 110–135 nm that express the exosomal makers CD9 and CD63. In addition, EVs were characterized using two different measurement techniques, NTA with NanoSight and cryo-TEM observations. Cryo-TEM images of EVs supported the results of NTA with NanoSight, in which EVs comprised a heterogeneous population. Thus, when measuring the particle properties of heterogeneous populations, such as EVs, proper and multifaceted analyses using adequate instruments are required.

## 5. Conclusions

The present study aimed to identify aHSC-tropic EVs by focusing on cell-to-cell interactions in the liver under pathological conditions, and by evaluating the isolation of hepatocyte-derived EVs and their uptake into aHSCs. The obtained results suggest that treatment of hepatocytes with PA causes the release of EVs with aHSC-tropism. The present results may help to develop an EV-based DDS that suppresses the transfer of drugs to hepatocytes and has targeted specificity to aHSCs to realize a definitive therapy for liver fibrosis. They also provide new insights into the involvement of steatotic hepatocyte-derived EVs in the regulation of physiological function, development of various diseases, and control of the cellular microenvironment. The results of the present study suggest that EVs derived from hepatocytes might not only be applied as novel DDS carriers for delivering therapeutic drugs specifically to aHSCs but also as biomarkers for liver fibrosis.

## Figures and Tables

**Table 1:** Characterization of EV-AML^CNT^ and EV-AML^PA^ using NTA and BCA assays.

Physical Properties	EV-AML^CNT^	EV-AML^PA^
Mean particle diameter (nm), NTA	115.7 ± 0.9	136.7 ± 7.1
Particle concentration (10^9^ particles/mL), NTA	3.26 ± 0.291	1.09 ± 0.124
Protein concentration (μg/mL), BCA	23.444	17.000
Amount of protein/particle (10^−9^ μg protein/particle)	7.191	15.596

NTA: nano-tracking analysis (NanoSight); BCA: bicinchoninic acid protein assay.

## Data Availability

m-RNA-sequencing data in the present study have been listed in the DDBJ Sequence Read Archive (https://www.ddbj.nig.ac.jp/dra/index.html accessed on 20 September 2022) under accession number DRA014859.
